# Some Biological Aspects of Bloodworm: *Chironomus pallidinubeculosus* Tokunaga, 1964 (Diptera: Chironomidae)

**DOI:** 10.21315/tlsr2024.35.2.11

**Published:** 2024-07-31

**Authors:** Patipat Tevapawat, Nisarat Tungpairojwong

**Affiliations:** 1 Department of Biology, Faculty of Science, Khon Kaen University, 16 Thanon Mittraphap, Nai Mueang, Mueang Khon Kaen District, Khon Kaen 40002, Thailand; 2 Applied Taxonomic Research Center, Khon Kaen University, 16 Thanon Mittraphap, Nai Mueang, Mueang Khon Kaen District, Khon Kaen 40002, Thailand

**Keywords:** Aquaculture, *Chironomus*, Life Cycle, Non-Biting Midge, Bloodworm

## Abstract

Some biological aspects of local bloodworms in Thailand were investigated. In this study, the larvae of one species of bloodworm, identified as *Chironomus pallidinubeculosus* Tokunaga 1936, were reared in plastic containers at 25°C and fed with fish feed solutions three days per week. The eggs sample were processed by histological and TEM techniques. Yield (g/m^2^), moisture, ash, crude protein, crude lipid, crude fibre and gross energy (KJ/g) were determined in reared larvae. The results showed that *C. pallidinubeculosus* larvae could survive and be bred in plastic containers, controlled laboratory conditions, and have four instars. The suitable physico-chemical parameters during rearing were low to moderate dissolved oxygen (1.18 mg/L–5.00 mg/L), electrical conductivity (462 μS–714 μS), and total dissolved solids (249 mg/L–378 mg/L). Moreover, adults had a high average number of eggs per one egg mass from 193.2 ± 49.99 to 331.86 ± 80.23 and an average hatchability of 90.69% to 94.49% during the 1st to 3rd generations, respectively. The life cycle of this study was approximately 19 to 23 days. Polylecithal and centrolecithal eggs were observed. After gelatinous mass removal, the egg was covered by non-regularly exochorionic jelly. The internal morphology of the egg is mainly composed of proteid yolk, lipids and dense granular. Larvae constituted 89.78% water; they had a high 15th day yield (g/m^2^), and the minimum area of larvae for mass culture was 1.2 cm^2^. The proximate composition analysis in reared larvae showed that crude protein, crude lipid crude fibre and gross energy were higher than its feed. The biology aspect study of the bloodworms found they were easy to culture; they should be considered a model organism for further ecology, nutrition and toxicology studies.

**Highlights**

- Bloodworms, *Chironomus pallidinubeculosus*, complete the life cyclein 17–23 days at 25**°**C. They have the possibility for further application or experimentation because they are simply cultured by luring natural bloodworms to lay their eggs on water inside plastic containers.
- Bloodworms lay a large number of eggs per lay (87–470 eggs), which are primarily made of a jelly-like substance that covers their mass to allow for floating. Their eggs are primarily yolk-containing and continue to shrink as they mature.
- Mass culture and rearing of bloodworms can be successfully accomplished with a fermented fish feed solution. Additionally, it improves bloodworm nutrition such as protein, lipid and crude fibre.

## INTRODUCTION

Bloodworms or chironomids are midge larvae classified in class Insecta, order Diptera and family Chironomidae. They are aquatic insects classified in the same order as mosquitoes and flies and are widely distributed along naturally running water, artificial ponds and canals. Their role in ecology is as prey to many larger predators such as fish and shrimp. They are distributed worldwide, with approximately 5,000 sp. described, but estimates of actual species range up to 20,000 (Ferrington *et al*. 2008). Chironomids are commonly called bloodworms because they have red pigment as blood, hemoglobin in their bodies to assist respiration (Walshe 1950; Osmulski & Leyko 1986; Burmester & Hankeln 2007; Sriariyanuwath *et al*. 2015). In Thailand, knowledge about the diversity of chironomids is scarce and limited. Cranston (2007) has reported the presence of 29 species and 15 genera of chironomid larvae associated with tsunami-impacted southwestern Thailand. According to the wide range distribution and considerable abundance, they were the most selected prey in nature by fish (De La Noüe & Choubert 1985; Tupinambás *et al*. 2015), shrimps (Albertoni *et al*. 2003; Zupo *et al*. 2016), and dragonfly larvae (Bo *et al*. 2012). The red colour of bloodworms or chironomids attracts fish as prey (Sahandi 2011), allowing fish to select prey on “living food” (Habib *et al*. 1992). Chironomid larvae and pupae prefer bottom-feeder organisms as food sources which is related to their high energy content (Armitage 1995). The nutritional quality of bloodworms is suitable for aquaculture diets (e.g., fish and shrimps) by evaluation from natural habitat (Bogut *et al*. 2007) and artificial diets (Habib *et al*. 1997; Maleknejad *et al*. 2014; Sahandi 2011; Habashy 2005). Additionally, the biology study of *C. circumdatus* was previously reported in Kuvangkadilok (1994) (known as *C. plumatisetigerus* Tokunaga 1945), reared in a temperature-controlled laboratory. Furthermore, *Chironomus riparius* and *C. sancticaroli* are a well-established model organism in various fields, such as eco-toxicology and ecology (Foucault *et al*. 2019; Corbi *et al*. 2019). Their egg study proceeded under a transmission electron microscope (Zissler & Sander 1973; 1977). Ultrastructural patterns in the ovarian follicles of adult *Culicoides punctatus*and *C. grisescens* (Diptera, Ceratopogonidae) were described (Filimonova & Brodskaya 1998). The aim of the study is to observe the biological aspects and conduct a nutritional study of local bloodworms in Thailand for further effective culture and toxicological study. The chironomid cultivation method was previously discussed by Credland (1973), Kuvangkadilok (1994), Habashy (2005), and Bhaduri *et al*. (2012). They used freshly laid egg masses, separate egg mass for hatching in a petri dish containing sterilised water, transferring first instar larvae to the rearing tray immediately after liberation. After that, the rearing experiments are monitored for an adequate supply of food material, aeration, and maintenance of photoperiod. The breeding method was previously discussed by Credland (1973) and Kuvangkadilok (1994). They used a cage and waited for the adult swarm, fertilise and oviposit egg mass on the Petri dish.

## MATERIALS AND METHODS

### Chironomid Collection and Species Isolation

Chironomid larvae were sampled by sweep net in a pond around Khon Kaen University, then carefully sorted from sediment by dropper (1 mL) and kept alive in a plastic container with tap water. They were temporarily kept at most 1 h before being put in an aquarium tank (width = 17 cm, length = 30 cm, height = 18 cm) with tap water (7 L) and covered with a fine net to prevent adults from escaping (Fonseca & Rocha 2004; OECD 2011) and the entry of other insects (Monika *et al*. 2016). An oxygen pump and air stones were added. Two mL of fish feed solution (27 g/L) was added daily, adapted from Credland (1973) and Habashy (2005). Fish feed solutions were prepared by using 1 L of tap water mixed with 27 g of fish feed flakes and incubated in a plastic bottle for at least 48 h. The physico-chemical water parameters during the egg mass collection period are shown in Table 1. Consequently, daily observation was made until the adult mates and deposits egg ropes. These eggs were collected, sorted, isolated and counted.

### Life Cycle Study

Egg ropes were put in plastic cups (diameter = 6 cm, depth = 4.5 cm) with tap water (120 mL) by one cup per egg rope, and newly hatched larvae were fed with 0.1 mL fish feed solutions (27 g/L) three times per week following Credland (1973). Investigation of the life cycle of chironomid larvae took place at 25°C under the photoperiod of 12: 12 (light: dark) at the Department of Biology, Faculty of Science, Khon Kaen University. All plastic cups were reared for three generations. Hatched eggs were counted. Three to five chironomid specimens were randomly collected every two days. All head capsule width of sample larvae was measured by an ocular micrometer on a light microscope to classify larval. Some physico-chemical elements of water parameters in a plastic cup was measured two times per week until adults emerged, including water temperature and dissolved oxygen (DO) (YSI 550A Dissolved Oxygen Instrument, USA), pH, electrical conductivity (EC) and total dissolved solids (TDS) (Hanna HI98129 Low-Ranged pH/Conductivity/TDS meter, Mauritius) APHA (2017). Furthermore, the air temperature was measured one time.

### Chironomid Identification

Larvae and adults from five plastic cups were randomly collected, preserved in 70% ethanol, and identified into species following Martin (2020) and Chaudhuri *et al*. (1992). The morphological characteristics of species identification included larvae mouthpart, especially mentum and mandible, larvae ventromental plates, adult wing and adult male genitalia.

### Egg Morphology Study

Five egg ropes or fertilised eggs from plastic cups in a life cycle study were randomly collected, and eggs from ropes were separated in tap water. Furthermore, unfertilised eggs were collected and separated from the female abdomen. Both separated eggs were fixed by absolute ethanol, put in a critical point dryer, attached to stub and gold coating, then observed external morphology under a Scanning Electron Microscope (SEM) (FEI, Model: Helios NanoLab G3 CX).

### Histological Study

Five egg ropes from 10% formalin were processed by histological techniques (H&E stain) following Barbosa *et al*. (2014). Then sample blocks were sectioned using a rotary microtome on a thin section (5 μm) and were put in the glass slide. After that, the morphology of cross and long-sectioned samples was observed under a light microscope (Olympus, Model: CH30).

### Ultrastructure Study

Five egg ropes from Karnovsky’s fixative (2% glutaraldehyde and 2% paraformaldehyde in 0.1 M sodium phosphate buffer pH 7.4) were processed following Hayat (2000). The embedding media was composed of a mixture as follows: 13 mL of Epon 812, 8 mL of DDSA, and 7 mL of NMA, then 16 drops of DMP-30. After embedding samples in the mold, the mold was placed in a hot air oven at 60°C for 48 h. The block samples were sectioned under ultramicrotome as thin section as 90 nm per section. The sectioned sample was put into four to six copper grids per block and stained with lead citrate for 1 min. After that, the grids were examined and characterised under Transmission Electron Microscope (TEM) (FEI, Model: TECNAI G2 20), especially their internal structure, following Zissler and Sander (1973; 1977), Gaino and Fava (1995) and Gautam *et al*. (2015).

### Mass Cultural Study

Chironomid eggs were obtained by putting plastic containers with tap water at the outdoor cultural laboratory, Department of Biology Faculty of Science, Khon Kaen University. Before the mass culture, egg ropes from the environment were preliminarily cultured in plastic containers (diameter = 33 cm, height = 11 cm), which followed and modified by Maleknejad *et al*. (2014). After this, chironomid adults were randomly collected and identified as by Martin (2020), and Chaudhuri *et al*. (1992). The plastic containers were settled as by Maleknejad *et al*. (2014) and 2 mL of fish feed solutions (27 g/L) were added twice per week as per Credland (1973). The experiment was conducted as four replicates under the photoperiod of 12: 12 (light: dark). The physico-chemical water parameters were measured on the 1st, 7th and 15th days including water temperature, pH, electrical conductivity, total dissolved solids, dissolved oxygen and five days of biochemical oxygen demand (APHA 2017). The productivity of each treatment as fresh weight (g/m^2^) of chironomid was measured on the 7th and 15th day following Maleknejad *et al*. (2014) and Habashy (2005).

### Nutritional Study

After the 15th-day productivity measurement, Chironomid larvae were continuously cultured and intensively collected and kept at −20°C weekly into at least 300 g fresh weight for nutrition analysis. The nutrition was measured in triplicate. Each treatment includes crude protein, crude fat, crude fiber ash and N-free extractive substances as a percentage, according to AOAC (1990). Therefore, the gross energy of chironomid larvae in each treatment was measured by the automatic adiabatic bomb calorimeter (KJ/g).

### Data Analysis

The head capsule width of all larvae was analysed by regression analysis, performed by the statistical programme SPSS version 26 (SPSS Inc., Chicago, IL, USA) to classify instar larvae.

## RESULTS

### Physico-Chemical Water Parameters

The physico-chemical water parameters during the observed life cycle are shown in Table 1. They can live in a wide range of DO with an average from 3.04 ± 1.01 mg/L to 3.55 ± 1.29 mg/L, neutral pH from 7.018 ± 0.13 to 7.148 ± 0.09, EC from 511.60 ± 57.12 μS/cm to 573.60 ± 80.14 μS/cm and TDS from 271.40 ± 29.64 to 304.20 ± 46.26 mg/L. Water parameters during the mass cultural study are shown in Table 4.

### Life Cycle

**Figure 1:**
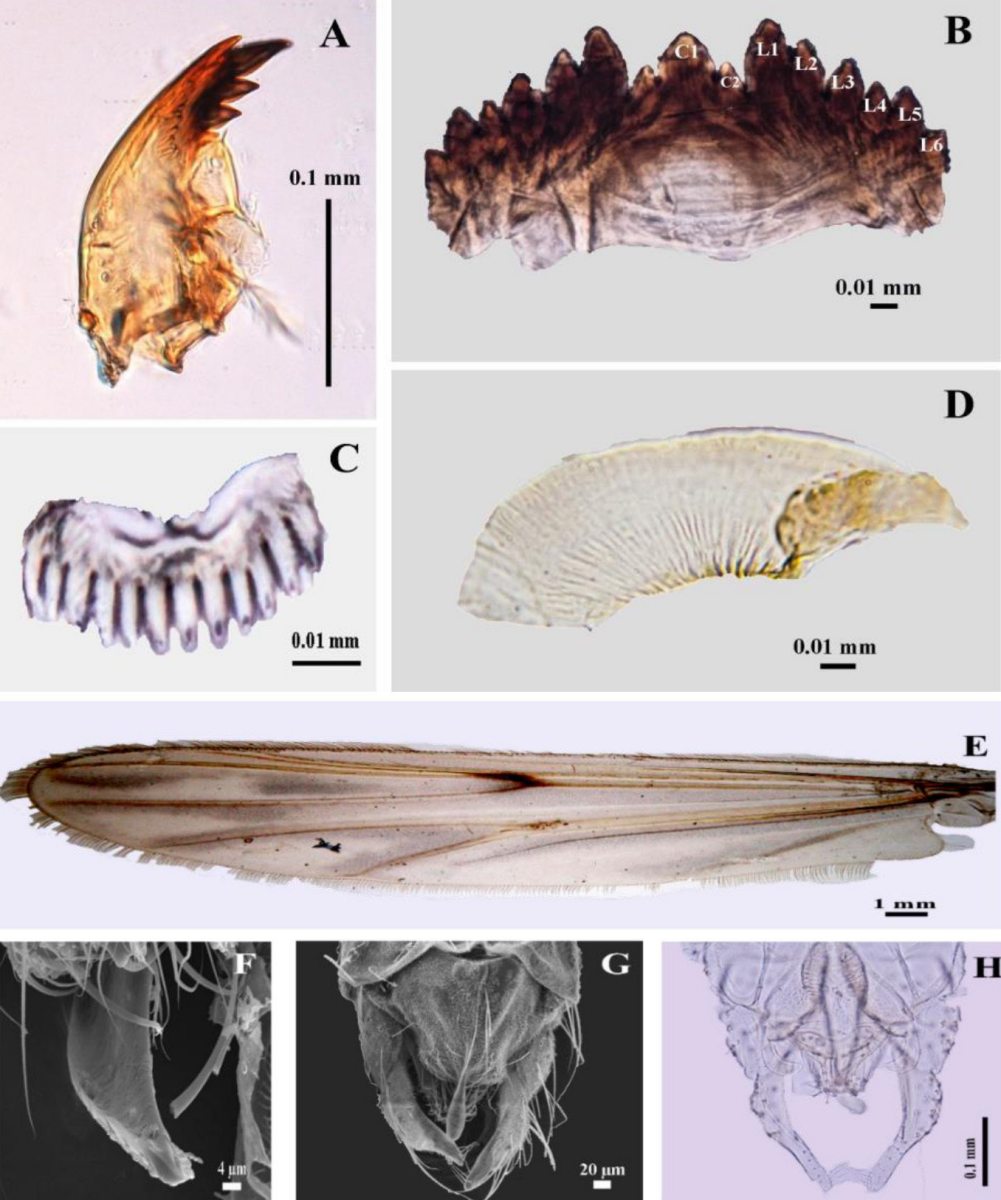
Morphological characteristics of *Chironomus pallidinubeculosus* to the identification. (A) mandible; (B) mentum; (C) pecten epipharyngis; (D) ventromentral plate; (E)wing; (F) anal point tip; (G) male genitalia in dorsal view under SEM with an anal point;(H)male genitalia in dorsal view with superior vorsella. Legend: central tooth (C); lateral tooth (L).

**Figure 2:**
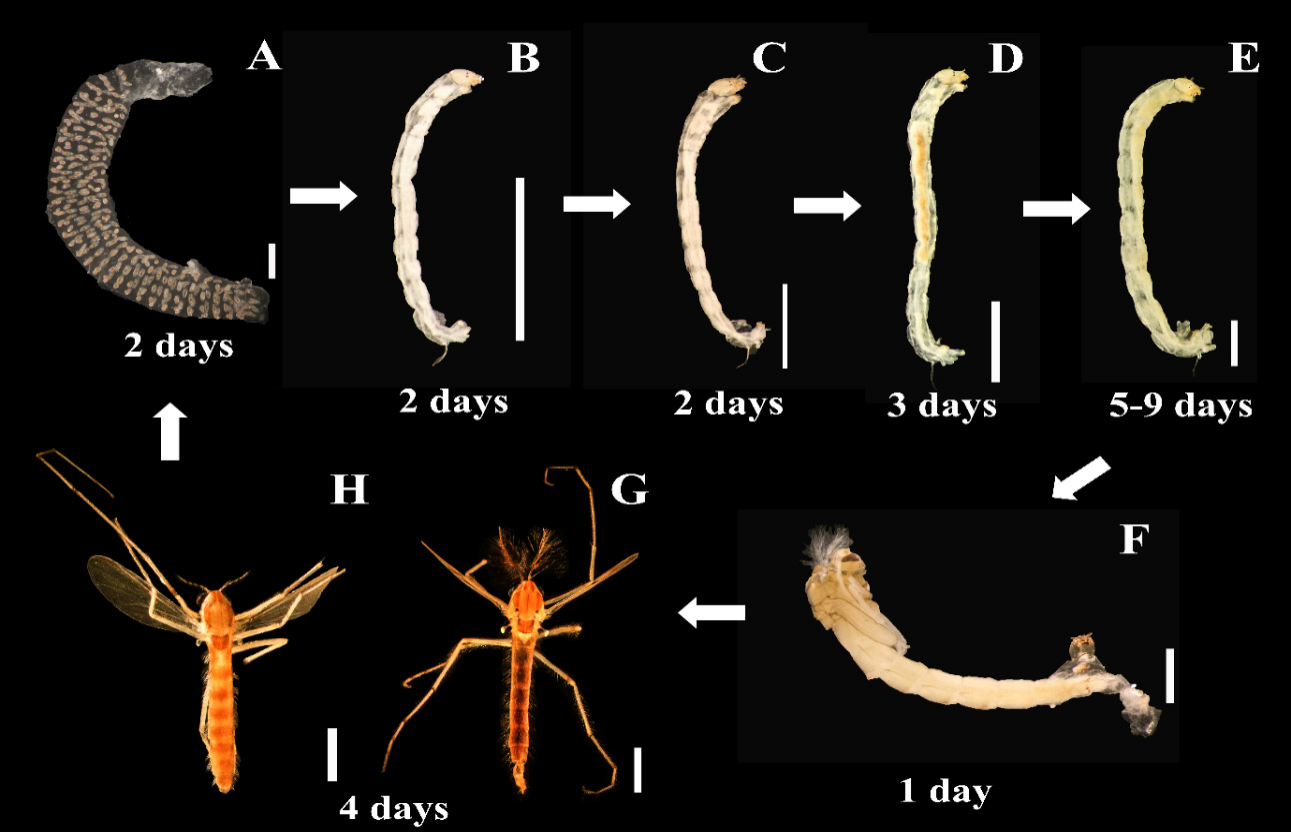
The life cycle and period of each stage of *Chironomus pallidinubeculosus* from egg to adult. (A) egg mass; (B) first instar; (C) second instar; (D) third instar; (E) fourth instar; (F) pupa; (G) male adult; and (H) female adult.

In this study, one species of bloodworm was identified as *Chironomus pallidinubeculosus* Tokunaga 1936. The character to identification is shown in Fig. 1. The life cycle of this study is approximately 19 to 23 days, and the character of every instar is shown in Fig. 2.

#### Egg

Eggs were laid in a gelatinous mass (length = 8.88 ± 2.23 mm, width = 1.44 ± 0.25 mm, *n* = 15), arranged as spiral inside similar to ropes with a cylindrical shape, attached substrate, and floatable at the water surface (Fig. 2A). The number of eggs per mass range from 83–470, and the range of average hatchability by 90.69%–94.49% (Table 3). All eggs hatched in 2 days.

#### Larva

**Figure 3:**
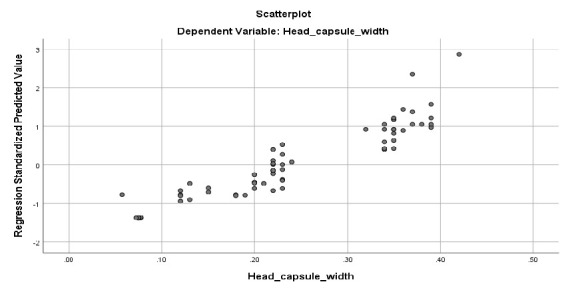
Plots of regression analysis. (A) head capsule width and (B) length.

Seventy-nine larvae samples were selected during the rear to measure head capsule width. *C. pallidinubeculosus* larvae have four instars followed by regression analysis as shown in Fig. 3 and Table 2, which correspond with the life cycle study.

1. **First instar**: They have a duration of two days. Early hatched larvae remainon gelatinous mass and then freely swim around. They are colourless and lack ventral tubes at the 10th tergile and anal gill at the 12th tergile (Fig. 2B). They build small cases using a mixture of food debris and their waste. Theirhead capsule width and length are 85 μm and 1.1 mm–1.14 mm, respectively(Table 2).
2. **Second instar**: They have a duration of two days. Their bodies are slightly red. Ventral tubes at the 10th tergile and anal gill at the 12th tergile are developed. Lack of swimming which only lives in cases (Fig. 2C). Their head capsule width and length are 130 μm–140 μm and 1.9 mm–2.4 mm, respectively (Table 2).
3. **Third instar**: They have a duration of three days. Their bodies are markedly red and larger than the second instar (Fig. 2C). Their head capsule width and length are 200 μm −240 μm and 3.5 mm–5.42 mm, respectively (Table 2).
4. **Fourth instar**: They have a duration of 5–9 days which is the longest duration in the larval period. Their bodies are red to dark red, elongated, cylindrical and slender. The larval head capsule is markedly smaller than the body. Ventraltubes at the 10th tergile and anal gill at the 12th tergile are well developed(Fig. 2D). The 12th abdomen at the posterior has well-developed claws. Theirhead capsule width and length are 340 μm–390 μm and 5.12 mm–8.55 mm, respectively (Table 2). Larva species identification is more complicated than with adults, by combining some mouthpart characteristics including 3rd inner tooth of the mandible separate and pale (Fig. 1A), a second central tooth (C2)of the mentum distinctly separate from the lateral tooth, and a fourth lateral tooth (L4) which has significantly reduced (Fig. 1B); pecten epipharyngis single pale has 12 to 14 sharp teeth (Fig. 1C); ventromentral plate has 29 to 36 striae (Fig. 1D).

#### Pupa

Compared with the larval period, they have a short duration of 1 day. They have plumose antennae. The early pupa remains on the bottom of the container, however, they rapidly swim to the water’s surface during emergence both during day and night (Fig. 2F).

#### Adult

They live for 4 days. Adult males and females show the simplest different characteristics in antennae and body size. The males have very plumose antennae and fusiform-slender body shapes (Fig. 2G). The females have pilose antennae and cylindrical body shapes (Fig. 2H). The adult male genitalia are very complicated as with other species. However, its hypopygium with curved anal point (Fig. 1F) superior vorsella is strongly curved at the tip, gonostylus is wider in the middle with 6 setae at its inner apical margin (Figs. 1G and 1H). Consequently, the specific characteristic for species identification was two cloudy dark spots or dark nebulae on wings particularly in cell R_4–5_, one linear spot in cell M_1+2,_ and a dark spot over the cross vein at R_s_ (Fig. 1E).

### Egg Morphology

**Figure 4:**
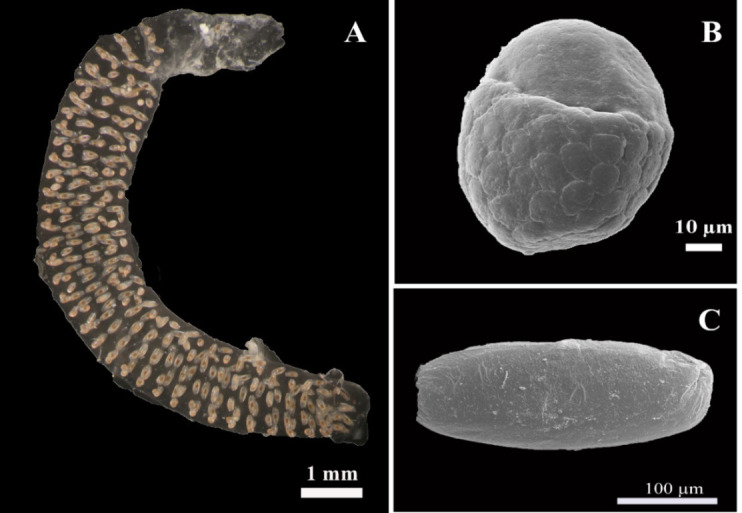
Eggs of *Chironomus pallidinubeculosus* (A) fertilised egg mass; (B) unfertilised egg under SEM; and (C) fertilised egg under SEM. (scale = 1 mm)

Egg ropes: Fertilised egg masses are covered by a transparent floatable jelly substance and one egg mass contains 83 to 470 eggs. Within the mass, the cords or strings may be arranged spirally, in horseshoe-like loops (Fig. 4A). The unfertilised egg: is an oval shape, three quarters has a small hexagonal plate pattern arranged outside (Fig. 4B). Fertilised egg: ellipse shape, rough surface and coated with a jelly substance (Fig. 4C).

### Egg Ultrastructure

**Figure 5:**
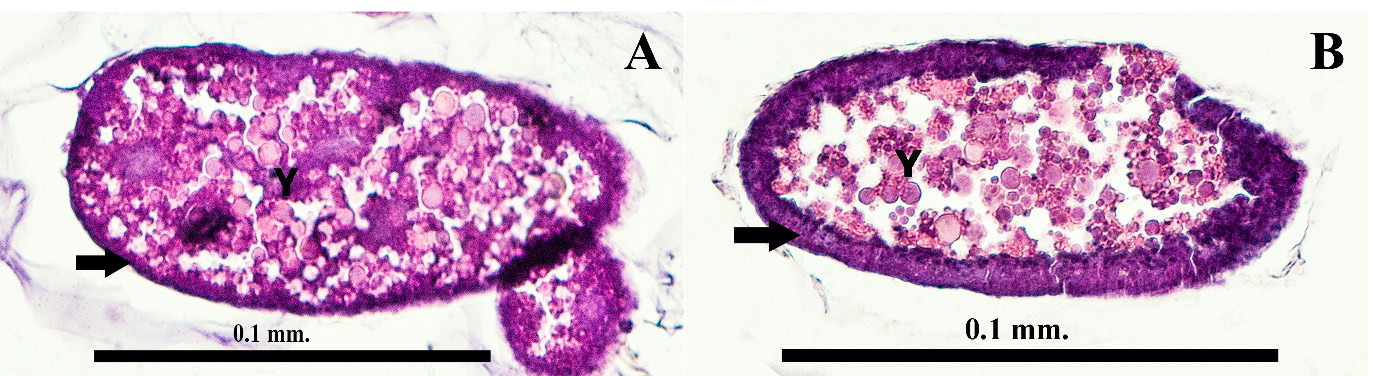
The section fertilised eggs of *Chironomus pallidinubeculosus* (A) cross-section at the early stage and (B) cross-section at the late stage. Legends: periplasm (arrow), yolk (Y).

**Figure 6.**
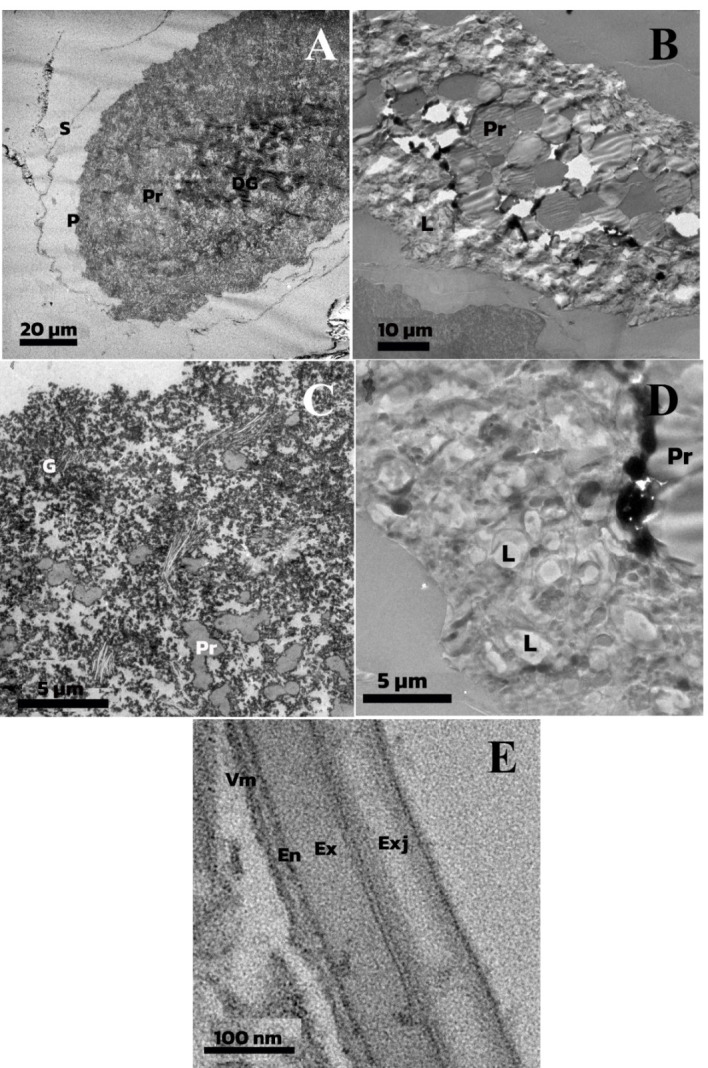
*Chironomus pallidinubeculosus* egg features under TEM. (A) early stage; (B) late stage; (C) early stage yolk; (D) late stage yolk; and (E) shell. S = shell, P = periplasm, Pr = proteid yolk, DG = dense granule, L = lipid, G = granule, Exj = exochorionic jelly, Ex = exochorion, En = endochorion, Vm = vitelline membrane.

Figs. 5 and 6 show a cross-section and long section of a fertilised egg from the H&E stain technique. They are divided into two stages early stage (Figs. 5A and 6A) and late stage (Figs. 5B and 6B). The different stages are indicated by the thickness of the periplasm and the density of the yolk. The eggs are classified as polylecithal and centrolecithal types by numerous yolks. The periplasm (Fig. 6A) is the yolk-free layer that covers yolk systems. The early stage of the egg (Fig. 6A) has distributed granules and proteid yolk with dense granular almost at the centre region (Fig. 6C). The late stage of the egg (Fig. 6B) is occupied by the yolk endoplasm (yolk system) which consists of a cytoplasmic network interspersed with proteid yolk bodies and lipid droplets (Fig. 6D). The shell structure of the fertilised egg is composed of exochorionic jelly (28 nm–73 nm) at the external layer, exochorion (60 nm–65 nm), endochorion (11 nm–14 nm), and vitelline membrane (Fig. 6E).

### Chironomid Productivity and Nutrition

The yield of chironomid grown in fish feed solutions at 15 days was 280.84 ± 55.80 g/m^2^ (Table 4). The number of chironomids is approximately 700 individuals in each plastic container (1 individual per area = 1.2 cm^2^). Chironomid has a percentage proximate composition including crude protein (54.16 ± 0.41%), lipid (5.92 ± 0.004%), fibre (5.03 ± 0.44%) and gross energy (17.44 ± 0.10 KJ/g) higher than fish feed solutions (Table 5).

## DISCUSSION

During life cycle observation and mass culture, the DO value was at least 3 mg/L and the pH value was 7 which they are within the permissible limit of the Surface Water Quality Standards of Thailand (National Environment Board 1994). Water parameters were in a high range for life cycle observation and mass culturing such as dissolved oxygen, electrical conductivity, and total dissolved solid. This indicates that *C. pallidinubeculosus* has adapted to a wide range of living factors. Walshe (1950), Osmulski and Leyko (1986) and Burmester and Hankeln (2007) explain that hemoglobin within chironomid enables the larva to maintain the active process of filter-feeding when insufficient oxygen is present. It acts in oxygen transport at severe shortages, thereby enabling continued respiratory irrigation and it greatly increases the rate of recovery from periods of low oxygen, making such recovery possible even under adverse respiratory conditions. In addition, the living behavior of chironomid in this study is mostly tube-like and its process of construction corresponds closely to the descriptions by Leathers (1922) and Oliver (1971). Larvae burrowed into the substratum and construct tubes with sediment and silk around the body. Larvae then appeared to wriggle through the tube. This behaviour allows the larvae to hide from predators and live under severe oxygen concentrations (Pinder 1986). EC (511–573 μS/cm) and TDS (271–304 mg/L) values during life cycle observation have the medium range and remain higher (Table 1). The main reason was the addition of fish feed each week and bacteria breakdown of organic matter under aerobic conditions. Also, the high EC and TDS values. Pinder (1986) discussed suggest that the rich organic matter is probably indicative of a positive correlation with chironomids.

The life cycle of the egg, larvae, pupa, adult period and fecundity of *C.pallidinubeculosus* in this study have similarity to Syrjämäki (1965), Bhaduri *et al*. (2012) and Toebae (2015), as shown in Table 6. On the contrary, head capsule width larvae in this study are distinctly smaller than Bhaduri *et al*.2012. Furthermore, Kuvangkadilok (1994) reared *C. circumdatus* which has a longer adult duration and head capsule width larvae slightly larger than *C.pallidinubeculosus*. Chaudhuri and Ghosh (1986) investigated the life cycle of *Kiefferulus harbatitarsis* and *K. calligaster* which have a longer larval period than *C.pallidinubeculosus*. The hatchability and fecundity in this study are up to 90% which is similar to Kuvangkadilok (1994). The larvae period of Syrjämäki (1965) was completed within 5 days at 28°C, which is the highest temperature among publications reviewed. The very fast growth of larvae suggests that temperature is one factor that influences the growth and development of *Chironomus* (Fischer& Rosin 1969) and *Kiefferulus* larvae (Chaudhuri *&* Ghosh 1986). The period of the fourth instar is prolonged, which might be due to the occurrence of maximum physiological changes in this stage (Chaudhuri & Ghosh 1986). Different species within the same reared temperature yielded different results.

Egg mass shapes and arrangements were the same as those described by Hinton (1981). The jelly substance is a mucoprotein, while Chironomidae chiefly consists of a polysaccharide. The jelly functions as a drought tolerant protector of the eggs when they are not in water (Hinton 1981). The coated jelly substance was classified by the distinct irregular thickness of the outermost layer as exochorionic jelly (Fig. 6E), which is the same as *Tribolium castaneum* (Gautam *et al*. 2015).

The chorion of fertilised chironomid egg was shown but not described in Zissler and Sander (1973; 1977). Chorion consists of two very distinct layers (King 1960; Cummings 1972; Mathew *&* Rai 1975; Pollard *et al*. 1986). Exochorion in chironomid was a smooth layer, dissimilar to reports from Tadkowski and Jones (1979), Hinton (1968) and Sahlen (1990). In mosquito egg, the “fibrous mesh” was identified as the exochorion in *Aedes aegypti* and *Culex pipens*. The space between the chorion supports the respiratory and gas exchange of embryos (Hinton 1981; King 1960; Gautam *et al*. 2015). Furthermore, the endochorion is of greater density than exochorion. King (1960) studied *Drosophila melanogaster* eggs and found the inner layer of the shell was dense when observed from an electron microscope. The chorion thickness was markedly different. Exochorion was thicker than endochorion, in agreement with Gaino and Fava (1995). The chorion thickness of some insect pests coleopteran and dipteran are different (Gautam *et al*. 2015).

Periplasm and yolk are classified by a random distribution of cell elements. The main composition of the yolk is proteid yolk and the accumulation pattern within the egg has a pattern similar to Zissler and Sander (1977). Yolk within chironomid is composed of proteid yolk particles, lipid droplets and accumulations of glycogen. The classification of proteid yolk and lipid was the lead citrate staining process. It sensitively binds to proteins causing dark or grey matter in electron observation. The lipid droplets are also globular and transparent as they are less sensitive to lead citrate staining. At the early stage of a fertilised egg, a dense granulated substance was observed and classified by dark amorphous molecule (Figs. 6A–6D). Yolk accumulation in the oocyte occurs simultaneously with the vitelline membrane synthesis by follicle cells (Filimonova & Brodskaya 1998). However, most of the protein yolk forms while the periplasm is filled with micropinocytotic invaginations and tubules derived from the oolemma (Mahowald 1972). Thicker periplasm and lower density of yolk indicate an advance in embryogenesis, as shown in Figs. 5A–5B and 6A–6B (Hinton 1981).

The reduction of total dissolved solid and electrical conductivity from the beginning of the culture was probably due to the use of organic matter by the midge larvae and the breakdown of nutrients by bacteria under aerobic conditions (Habib *et al*. 1997). Fish feed solutions were effective in chironomid grown at 15th days (Table 4). Armitage (1995) and Bogut *et al*. (2007) showed protein, lipid and ash were rich amounts in chironomid larvae. The energy level in this study is similar to that seen in Yurkowski and Tabachek (1979; as cited in Armitage 1995) and lower than Wissing and Hasler (1968; 1971), Cummins and Wuycheck (1971) and De la Noüe and Choubert (1985).

Female midges were attracted to oviposit by fermented feed at wide-range habitats. Strenzke (1959; as cited in Creadland 1973) noted natural sites at which he found the eggs. For example glass, wood, cork, non-toxic plastics, filter paper and banks of natural aquatic and plant leaves. In this study, females oviposit their egg at the inside container margin, above the water surface.

The high yield of larvae as density in this study (1 individual per 1.2 cm^2^) was similar to Credland (1973). The maximum population *C. riparius* larvae in the tank was 1 individual per 2 cm^2^. *C. tentans* requires 5 cm^2^ (Beermann 1953 as cited in Credland 1973). *C. thummi* 4 cm^2^ (Strenzke *&* Neumann 1960 cited in Creadland 1973). Rasmussen (1985) admits that density can have a strong relationship to the growth of chironomid larvae. The available surface area in the upper layer of sediment for building their tubes may have been a limiting factor (Biever 1971). The higher survival rate of chironomid in limited areas during mass culture can be explained by Walshe (1948). The high metabolic rate of chironomid larvae is only maintained in fully aerated water; oxygen condition has an effect on larval energy metabolism, with low oxygen or anoxia condition causing lower survival rates in newly hatched larvae. The younger larvae have weaker adaptation to anoxia conditions than fully grown larvae (Hamburger *et al*. 1995).

The fish feed solution in this study had higher gross energy than fish feed from Habashy (2005) but percentages of protein, lipid and carbohydrate were lower. Cultured chironomid larvae had higher protein percentages than Habashy (2005). Carbohydrate percentage was similar and lower in percentage of lipid and ash. In addition, chironomid grown in palm oil mill effluent from Habib *et al*. (1997) has a similar proportion of crude protein as this study but a markedly higher percentage of crude fat.

Bogut *et al*. (2007) suggest that *Chironomus plumosus* larvae represent potentially suitable nutrition for farmed fish because of their high total lipid content and essential fatty acids which have a beneficial effect on fish health, growth, and development. The live food industry could establish the cultivation of chironomid larvae for use as fish feed. Their relatively high digestibility and the numerous growth promoters means that chironomid larvae represent a rich energy source for fish (De La Noüe *&* Choubert 1985). Culturing chironomid with fish feed avoids problems of starvation and putrefaction. Biever (1971) and Credland (1973) report that fish feed enhances the growth rate in a laboratory culture of many species of chironomid larvae. Further, chironomid gets rid of dissolved organic matter from fish feed solution by ingestion. The present results suggested that fish feed was an effective food for chironomid larvae production which increases sufficiency production for feeding ornamental and economical aquatic animals.

## CONCLUSION

This study of some of the biological aspects of *C. pallidinubeculosus* shows high hatch ability (up to 90%), fecundity (approximately 300 number per egg mass), easy availability and culturing, rapid life cycle (complete cycle in 27 days). Offering a source of high nutrition and a wide array of living factors *C. pallidinubeculosus* is suitable for many purposes including aquatic toxicology model organism, ecology and living prey for commercial aquatic organisms in Thailand.

## Figures and Tables

**Table 1:** Mean ± SD values of some physico-chemical parameters of water during the rearing of bloodworms.

Parameters/ Generation	Dissolved oxygen (mg/L)	pH	Water temperature (°C)	Electrical conductivity (μS/cm)	Total dissolved solid (mg/L)
Initial (from broodstock)	3.77 ± 0.16 (3.65–3.95)	7.55 ± 0.44 (7.15–8.02)	26.20 ± 0.26 (26.00–26.50)	408.00 ± 31.19 (379–441)	215.67 ± 14.57 (202–231)
1st	3.29 ± 1.6 (1.18–5.00)	7.11 ± 0.06 (7.04–7.18)	23.48 ± 0.57 (22.5–23.8)	511.60 ± 57.12 (462–610)	271.40 ± 29.64 (249–323)
2nd	3.55 ± 1.29 (1.68–4.55)	7.018 ± 0.13 (6.83–7.16)	22.36 ± 0.47 (21.8–23.1)	535.80 ± 71.55 (483–659)	282.20 ± 39.92 (253–351)
3rd	3.04 ± 1.01 (1.76–4.5)	7.148 ± 0.09 (7.01–7.25)	24.06 ± 0.32 (23.8–24.5)	573.60 ± 80.14 (514–714)	304.20 ± 46.26 (272–378)

*Note*: Data in brackets represents a range of water parameters.

**Table 2:** Growth and development of *Chironomus pallidinubeculosus* larvae.

Instar	Larval period (days)	Head capsule width (range in μm)	Length (range in mm)
I	2	85 (20)	1–1.14
II	3	130–140 (12)	1.9–2.4
III	3	200–240 (26)	3.5–5.42
IV	5–9	340–390 (21)	5.12–8.55

*Note*: Data in bracket represents a number of chironomid samples.

**Table 3:** Fertility and hatchability of *Chironomus pallidinubeculosus* egg masses.

Generation	Number of eggs	The average number of eggs	Average hatchability (%)
F_1_	83–253	193.2 ± 49.99 (10)	93.99 ± 2.82 (5)
F_2_	191–470	304 ± 97.61 (11)	90.69 ± 4.82 (6)
F_3_	222–392	331.8 ± 80.23 (7)	94.49 ± 6.06 (5)

*Note*: F1 = first generation; F2 = second generation; F3 = third generation; Data in bracket represents the number of egg mass.

**Table 4:** Mean ± SD values of some physico-chemical parameters of water during harvest day and yield of chironomid larvae grown in fish feed solutions (g/m^2^).

Period of harvest (day)	Parameters					The yield of chironomid larvae grown in fish feed solutions (g/m^2^)

	Dissolved oxygen (mg/L)	pH	Water temperature (°C)	Electrical conductivity (μS/cm)	Total dissolved solid (mg/L)	
1	4.14 ± 0.50 (3.63–4.57)	7.39 ± 0.03 (7.35–7.41)	27.20 ± 0.14 (27.0–27.3)	998.25 ± 179.31 (855–1258)	517.50 ± 79.24 (441–627)	-
7	4.38 ± 0.31 (4.20–4.84)	7.49 ± 0.07 (7.41–7.57)	27.28 ± 0.05 (27.2–27.3)	888.50 ± 127.99 (750–1060)	471.25 ± 67.72 (398–562)	154.56 ± 14.13 (135.98–170.21)
15	5.77 ± 0.37 (5.47–6.24)	7.78 ± 0.02 (7.76–7.81)	26.45 ± 0.17 (26.3–26.7)	919.50 ± 155.59 (747–1125)	493.00 ± 75.02 (418–597)	280.84 ± 55.80 (223.83–354.56)

*Note*: Data in bracket represents a range of water parameters.

**Table 5:** Mean ± SD values of proximate composition (%) in fish feed solutions and chironomid larvae grown in fish feed solutions.

Proximate composition (%)	Fish feed solutions	Chironomid larvae are grown in fish feed solutions
Moisture	78.84 ± 0.62	89.78 ± 0.34
Ash	19.26 ± 0.12	19.81 ± 0.03
Crude protein	29.60 ± 0.17	54.16 ± 0.41
Crude lipid	2.81 ± 0.03	5.92 ± 0.004
Crude fiber	3.67 ± 0.06	5.03 ± 0.44
Nitrogen free extract (NFE)	44.11 ± 0.18	15.08 ± 0.84
Gross energy (KJ/g)	16.52 ± 0.08	17.44 ± 0.10

**Table 6:** Comparison of life cycle study among oriental species.

Species	Temperature (°C)	Egg (days)	1st instar (days)	2nd instar	3rd instar	4th instar	Pupa	Adult	Fecundity (no. of eggs)	References
*Chironomus pallidinubeculosus*	25	2	2	3	3	5– 9	1	4	83–470	This study
*C. pallidinubeculosus*	25	2–3	3	2	4	6– 8	1– 2	4– 5	500	Bhaduri *et al*. (2012)^a^
*C. pallidinubeculosus*	-	-	3–5	2–3	3–4	7–8	1	-	-	Toebae (2015) b
*C. circumdatus*	25	3–4	2–3	2	2	7– 10	1	7 – 8	55–724	Kuvangkadilok (1994) c
*Kiefferulus harbatitarsis*	25	2–3	5–6	6–7	7–9	13–16	2	1–2	-	Chaudhuri & Ghosh (1986)
*K. calligaster*	25	2–3	2–3	5–7	8–9	12–15	2–3	1–2	-	Chaudhuri & Ghosh (1986)

*Note:*

a Bhaduri *et al*. (2012) identified as *C. striatipennis* which synonym of *C. pallidinubeculosus;*

b Toebae (2015) identified as *C. calipterus* which synonym of *C. pallidinubeculosus;*

c Kuvangkadilok (1994) identified as *C. plumatisetigerus* which synonym of *C. circumdatus*.
